# Lower trapezius tendon transfer for irreparable rotator cuff injuries: a scoping review

**DOI:** 10.1016/j.xrrt.2022.08.006

**Published:** 2022-09-30

**Authors:** Veeral Desai, Thomas Stambulic, Parham Daneshvar, Ryan T. Bicknell

**Affiliations:** a School of Medicine, Queen's University, Kingston, Ontario, Canada; b Department of Othopaedic Surgery, Queen’s University, Kingston, Ontario, Canada

**Keywords:** Rotator cuff tear, Tendon transfer, Lower trapezius, Massive cuff tear, Scoping review, Orthopedic surgery

## Abstract

**Background:**

Rotator cuff tears are a common source of shoulder pain and dysfunction. An irreparable rotator cuff tear poses a particular treatment challenge. There have been few studies reporting the outcomes of lower trapezius tendon (LTT) transfer for irreparable rotator cuff injuries. Therefore, the purpose of this review is to summarize the postoperative functional outcomes and complications of patients undergoing a LTT transfer for massive irreparable rotator cuff injuries.

**Methods:**

A scoping review was performed using the Medline, Embase, Cochrane Central Register of Controlled Trials, and Google Scholar databases with the search terms “trapezius” AND “transfer.” Of 362 studies included for initial screening, 37 full-text citations were reviewed, with 5 studies meeting all the inclusion criteria to be included in the review. Two reviewers extracted data on study design, patient demographics, surgical technique, functional outcomes, range of motion (ROM), and complications for each study according to the predefined criteria.

**Results:**

Improvements in the preoperative to postoperative functional status, identified using the Disabilities of the Arm, Shoulder, and Hand (50.34 to 18), The American Shoulder and Elbow Surgeons Score (48.56 to 80.24), Visual Analog Scale (5.8 to 1.89), Single Assessment Numeric Evaluation (34.22 to 69.86), and Subjective Shoulder Value (52.24 to 77.66), were evident across all 5 studies. Preoperative to postoperative increases in ROM were seen for flexion (85 to 135), external rotation (18 to 52), and abduction (50 to 98). The overall complication rate was 18%, with seroma formation (8%) as the most common postoperative complication.

**Discussion/Conclusion:**

Our analysis showed that LTT transfer improved postoperative function, ROM, and pain for patients with irreparable rotator cuff tears with an overall complication rate of 18%. Future controlled studies are required to directly compare LTT transfer to other tendon transfers and other surgical techniques for irreparable rotator cuff tears.

Rotator cuff tears are a common cause of shoulder dysfunction and pain.^25^ Management for rotator cuff tears includes both nonsurgical and surgical options depending on the clinical context, with mixed evidence supporting surgical vs. nonsurgical management.^18^^,^^20^ Massive rotator cuff injuries are described as defects >5 cm or tears involving more than 1 tendon.^9^^,^^14^ Massive tears are often irreparable; however, some may be managed with advanced arthroscopic techniques and mobilization.^4^ Criteria for massive irreparable rotator cuff tears preoperatively on magnetic resonance imaging (MRI) include a fatty degeneration index >3 for the supraspinatus and fatty degeneration index >2 for the infraspinatus with a coronal oblique tear distance of >31 mm and a sagittal oblique tear distance of >32 mm.^26^ Additional MRI findings suggestive of irreparable rotator cuff tears include an increased inferior glenohumeral distance and tendon retraction at or beyond the glenoid.^5^ A definitive assessment of irreparability is made intraoperatively after tendon mobilization.

Irreparable rotator cuff tears are often managed nonsurgically, especially in patients at high risk of surgical complications and in those with decreased functional demands or mild pain and/or shoulder dysfunction. For patients in whom nonsurgical management fails or those who elect for initial surgical management, multiple surgical options are available. These include debridement with partial repair, reverse total shoulder arthroplasty, subacromial spacer insertion, superior capsular reconstruction, and tendon transfers. Latissimus dorsi, pectoralis major, and lower trapezius tendon (LTT) transfer have all been used as tendon transfers for the management of irreparable rotator cuff tears.

The LTT transfer has recently gained popularity to treat irreparable rotator cuff tears.^1^ The main objective is to decrease pain and improve strength and function to the shoulder joint. Biomechanical studies have shown that LTT transfer maximizes external rotation and recreates more normal glenohumeral kinematics and reactive forces.^13^^,^^16^ The procedure may be performed in an open or arthroscopically assisted manner.^7^^,^^8^^,^^21^^,^^22^^,^^24^ There is some variability in techniques, but the LTT is first detached from its insertion at the scapular spine and mobilized away from the middle trapezius.^7^ The LTT is then augmented, usually with an Achilles allograft. For arthroscopically assisted reattachment, the newly augmented tendon is pulled through the anterolateral port or moved through a subcutaneous tunnel and anchored into the anterosuperior or anterolateral tuberosity. Additional procedures including biceps tenodesis/tenotomy, partial cuff repair, and subacromial decompression may also be concomitantly performed during the LTT transfer.

The primary indications for using the LTT technique in the setting of irreparable massive posterosuperior rotator cuff tears include persistent pain and shoulder dysfunction with limited forward elevation and external rotation, concomitant subscapularis tears, and previously failed rotator cuff repairs.^8^^,^^22^^,^^23^ MRI findings which indicate massive irreparable tears suitable for LTT repair include Goutallier grade ≥3 for supraspinatus and ≥2 for infraspinatus, Patte grade ≥2, and a acromiohumeral interval of <7 mm.^23^ Contraindications to performing this procedure include severe glenohumeral arthritis, trapezius dysfunction or paralysis, deltoid or subscapularis deficiency, and advanced age.^23^ Clinical outcomes of LTT transfers for irreparable rotator cuff tears remain limited, with a systematic review by Clouette et al in 2020 including only 2 clinical studies on the topic.^2^ Therefore, the purpose of our scoping review is to characterize the indications, functional outcomes, and complications of patients undergoing LTT transfer for irreparable rotator cuff tears.

## Materials and methods

### Study design

A scoping review was performed to evaluate the literature and identify knowledge gaps in the use of LTT transfer for irreparable rotator cuff injuries. This review combines both qualitative and quantitative properties via a comprehensive search strategy and standardized study selection and evaluation. Due to the heterogeneity between studies and the limited sample size, combined with the lack of comparison groups in all but 1 study, no meta-analyses were performed.

### Selection criteria

Studies were included if the following inclusion criteria were met: (1) publication after the year 2000; (2) the use of human subjects; (3) age > 18 years; (4) LTT transfer for irreparable rotator cuff tears. Exclusion criteria included (1) non-English language; (2) the use of cadaveric subjects; (3) publication in the form of an abstract, letter, editorial, or review article; (4) LTT transfer for indications other than irreparable rotator cuff repair (ie, brachial plexus injury).

### Search strategy

MEDLINE, EMBASE, Cochrane Central Register of Controlled Trials, and Google Scholar databases were searched using the terms: “trapezius” AND “transfer”. A search algorithm is outlined in Supplementary Appendix S1.

### Study selection

**Figure 1:**
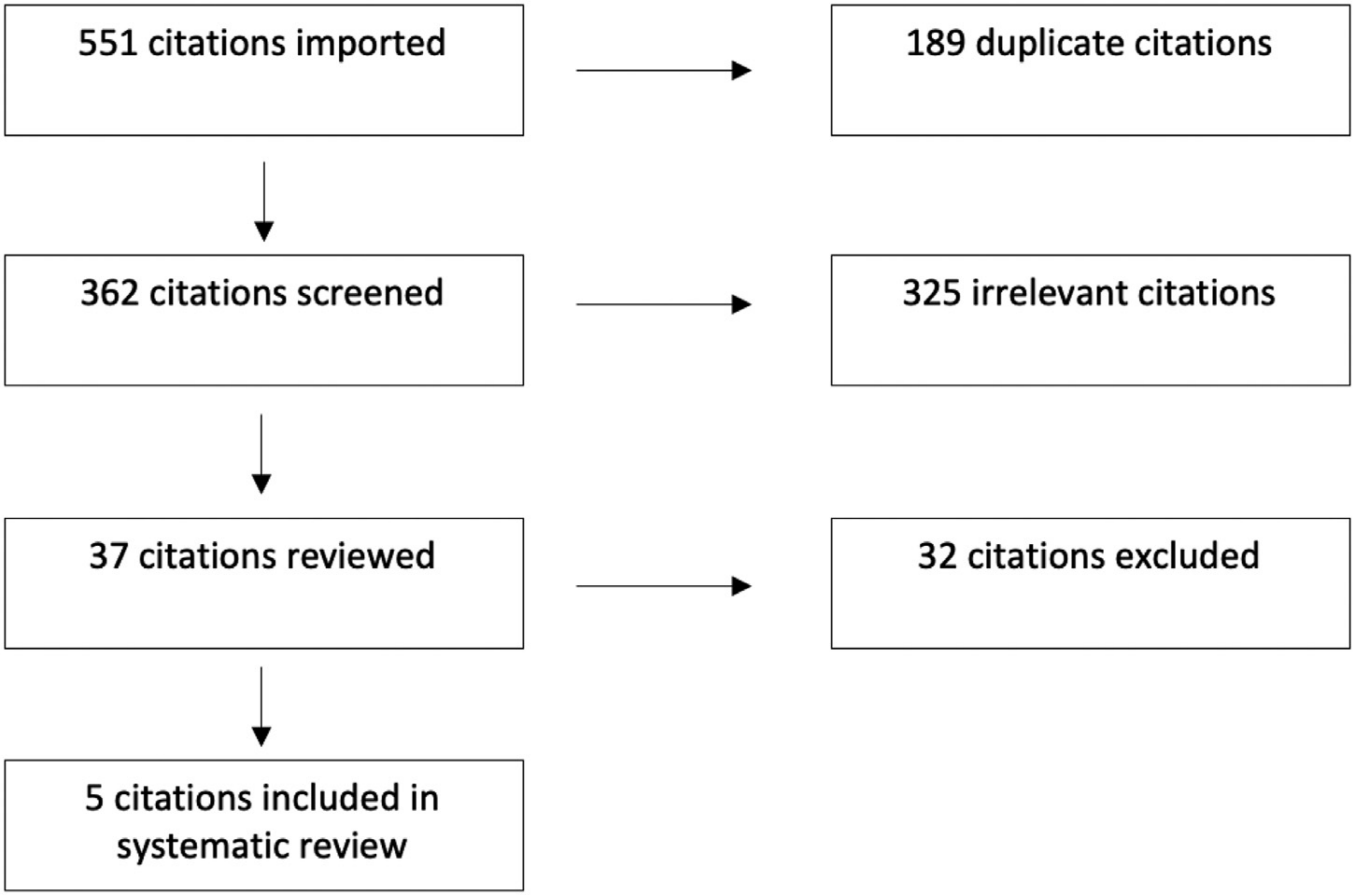
Preferred Reporting Items for Systematic Reviews and Meta-Analyses study-selection process.

The article selection was performed over 2 rounds by 2 reviewers (V.D. and T.S.) using the Covidence platform. During the first round, selection was based on the review of titles and abstracts. To be as inclusive as possible, an article was carried forward to the next stage if either reviewer thought that the study was appropriate. In the second round, final study selection was based on full-text review using the inclusion criteria. Duplicate studies were eliminated at the beginning of the process, using the Covidence software (Veritas Health Innovation Ltd, Melbourne, Australia). Consensus was reached for final article inclusion through a discussion among the investigators. The visual outline of this process can be viewed in Figure 1.

### Data extraction

Two reviewers manually extracted data from the 5 studies included in the scoping review. Data pertaining to study design, patient demographics, rotator cuff injury details, surgical indications, surgical technique, preoperative and postoperative functional outcomes, preoperative and postoperative range of motion (ROM), and complications were extracted for each study. Data were confirmed through agreement between all reviewers and are included in Table I, Table II, Table III, Table IV, Table V.

**Table I:** Study design and details of surgical technique.

Study	Study type	Surgical technique	Rehabilitation protocol	Follow-up (mo)
Elhassan et al, 2016^8^	Retrospective case review	Exposure of posterior-superior rotator cuff through osteotomy in the middle deltoid. Upper subscapularis and teres minor tears were repaired if present. Tenotomy and tenodesis were performed if the biceps was present and diseased. Achilles tendon allograft used to prolong the lower trapezius. A #2 nonabsorbable suture placed in Krakow fashion into tendinous and musculotendinous portions of lower trapezius then passed into the Achilles tendon. Multiple nonabsorbable #2 Orthocord (DePuy Synthes, Raynham, MA) sutures reinforced the repair. Endobuttons (Smith & Nephew, Inc., Andover, MA) were occasionally used to reinforce the repair. If impingement of spinal accessory was seen, 1-2 cm of the medial spine of the scapula was excised to avoid nerve impingement.	Patients were placed in a custom-made shoulder spica brace in 30 degrees abduction and 50 degrees external rotation for 8 weeks. From weeks 8 to 12, patients completed active assisted ROM exercises in all directions except internal rotation. After this, patients were allowed full range of motion and gentle strengthening exercises. Patients returned to unrestricted activities after 6 mo.	Mean: 47 Range: 24-73
Valenti and Werthel, 2018^22^	Prospective case series	The lower trapezius was harvested using a vertical posterior approach with reinforcement of nonabsorbable sutures placed into the tendinous and musculotendinous portions using a Krackow technique. The 20-cm semitendinosus autograft was harvested with reinforcement using a braided nonabsorbable suture with the Krackow technique. Fixation was done using 2 techniques: 1. Semitendinosus graft sutured medially to the tendon of the lower trapezius in a Pulvertaft fashion. A cortical button was then sutured to the lateral free end of the graft and shuttled into the bone tunnel under lateral arthroscopic visualization. The strands of the cortical button were tightened to introduce 2-3 cm of the graft into the tunnel. 2. Semitendinosus tendon was fixed by arthroscopy at the level of the infraspinatus footprint with 2 or 3 anchors. The medial stump of the graft was pushed to the subposterior deltoid space following the direction of the infraspinatus to reach the medial tubercle of the scapula. A Krackow suture placed in the trapezius and semitendinosus tendon reinforced the repair. In 4 patients, the lower trapezius transfer was combined to a latissimus dorsi transfer.	Custom-made shoulder brace in 30 degrees of abduction and 30 degrees of external rotation continuously for 6 weeks and only at night following 4 weeks. At 6 weeks, patients started pain-free passive and active assisted ROM exercises in elevation and external rotation. At 3 mo, full active ROM exercises were started with strengthening. Full return to unrestricted activities at 6 mo.	Mean: 24 Range: 18-36
Elhassan et al, 2020^7^	Retrospective case review	Lower trapezius tendon harvested before arthroscopic portion. Tendon prepared using nonabsorbable #2 suture in Krakow fashion. Lateral port served for visualization with anterolateral and anterior ports used for working. Subacromial decompression was performed with debridement of irreparable portions of rotator cuff. Achilles allograft prepared with 2 Krakow sutures into the calcaneal end. Allograft anchored into tuberosity with multiple suture anchors in the anterior aspect of the supraspinatus footprint medially and laterally. Multiple cycles of internal and external rotation were used to increase allograft tension. Partial rotator cuff repair, if possible, to the graft was performed in 22 patients and a tenotomy (n=4) or tenodesis (n=13) if biceps tendinitis or tear was present.	0 to 6-8 weeks: immobilization in a custom external rotation brace with shoulder maintained in 40-60 degrees of external rotation 6-8 to 12 weeks: passive, active-assisted and then active ROM with internal rotation limit to 0 degrees 12 to 16 weeks: gradual removal of passive and active internal rotation limit; return to most daily activities 16 weeks to 6 mo: gradual strengthening without motion limits 6 mo: return to full unrestricted activities	Mean: 14 Range: 6-19
Woodmass et al, 2020^24^	Retrospective case review	The LTT was harvested through a 6- to 8-cm incision over its insertion on the medial scapular spine. Two nonabsorbable running sutures were placed on either side of the Achilles tendon allograft, which was secured to the anterolateral aspect of the greater tuberosity. The allograft and LTT were secured with a Pulvertaft weave.	NA	Mean: 22 ± 10 Range: NA
Stone et al, 2021^21^	Retrospective case review	The open procedure (N = 9) involved a medial approach to dissect the LTT. An Achilles allograft was sutured to the lower trapezius using a Pulvertaft weave with multiple nonabsorbable sutures. The greater tuberosity was prepared with a bur, and the graft was stitched with multiple locking stitches from two 5.5-mm suture anchors placed medially on the footprint. A double-row construct was created using two 4.75 knotless suture anchors placed laterally. The arthroscopy-assisted procedure (N = 6) used a direct approach to the lower trapezius. A diagnostic arthroscopy was performed, and the tuberosity was prepared using burr and two 5.5-mm suture anchors placed on the medial aspect. The Achilles tendon allograft was prepared using a series of locking stitches into the tendon. The 2 suture limbs were tied to another at the medial aspect of the graft which was shuttled into its final position on the greater tuberosity with sutures tied arthroscopically. Two additional suture anchors were placed at the lateral footprint to complete a double-row repair. The graft was tensioned and sutured to the native trapezius in a Pulvertaft weave with multiple nonabsorbable sutures.	The shoulder was placed in a gunslinger abduction brace at 30 degrees abduction and 30-60 degrees external rotation for 6 weeks. Supine FE exercises were started 2 weeks after surgery. Physical therapy was initiated 6 weeks postoperatively, starting with ROM and restricting any cross-body adduction until week 12. At 12 weeks, rotator cuff strengthening began with scapular conditioning. Isotonic strengthening at 4 mo and return to full unrestricted activities at 6 mo.	Mean: 24 Range: 12-39

*ROM*, range of motion; *LTT*, lower trapezius tendon; *NA*, not available; *FE*, forward elevation.

**Table II:** Patient baseline demographics.

Study	Patient demographics	Baseline function	Type/degree of rotator cuff tear	Prior shoulder surgery	Details of previous surgeries
Elhassan et al, 2016^8^	N = 33 Male: 27 (82%) Female: 6 (18%) Age: 53 (31-66)	All patients reported preoperative repetitive lifting activities of 10 lbs or greater. Thirteen patients reported heavy lifting activities.	All patients had at least 2 full-thickness rotator cuff tears involving the supraspinatus and infraspinatus with Goutallier grade 3 or 4 and retraction of the torn tendon medial to the level of the glenoid. Patients all had acromiohumeral distance of less than 5 mm before surgery with no acetabulization. Ten patients had teres minor tear; 11 patients have evidence of fatty atrophy in the teres minor. Thirteen patients had tear of the upper part of subscapularis, but none had full-thickness tear or fatty atrophy of the subscapularis. Thirteen shoulders showed minimal arthritic changes of the glenohumeral joint. Gross arthritic changes were absent in all patients.	N = 22 (67%)	Twenty-two patients had attempted full or partial rotator cuff repair through open or arthroscopic technique. Three patients had 2 prior repairs, and 2 patients had 3 prior repairs. Ten patients had prior nonrotator cuff procedures including biceps tenotomy/tenodesis, subacromial decompression, acromioclavicular joint resection, and labral repair.
Valenti and Werthel, 2018^22^	N = 14 Male: 8 (57%) Female: 6 (43%) Age: 62 (50-70)	NA	All patients had a massive irreparable tear of the posterosuperior rotator cuff with atrophy of the teres minor and infraspinatus and fatty infiltration > grade 2 Goutallier classification. No patients had glenohumeral arthritis, pseudoparalysis, deltoid palsy, or an associated subscapularis tear > grade 2 Lafosse classification.	N = 9 (64%)	Nine patients had prior arthroscopic rotator cuff repair.
Elhassan et al, 2020^7^	N = 41 Male: 30 (73%) Female: 11 (27%) Age: 52 (37-71)	All patients were active with a desire to regain active shoulder use involving shoulder-level or overhead activities.	All patients had full-thickness tears involving the supraspinatus and infraspinatus with Goutallier grade >2 fatty infiltration, tendon length <1 cm, tendon retracted to the level medial to the glenoid, or Patte stage 2+. Nineteen patients had pseudoparalysis. Eight patients had teres minor fatty infiltration grade 3 or 4. Twenty patients had repairable subscapularis tear on the upper third of tendon. Five patients had repairable subscapularis tear on upper two-third of tendon. Ten patients had mild glenohumeral arthritis. Twenty-nine patients had Hamada grade 1, 2 patients had Hamada grade 2, and 2 patients had Hamada grade 3.	N = 33 (80%)	Twenty-seven patients with previous rotator cuff repair through an open or arthroscopic technique. Nine patients had 2 prior repairs, and 3 patients had more than 2 previous repairs. Six patients had a prior shoulder surgery not involving rotator cuff repair.
Woodmass et al, 2020^24^	N = 8 Male: 5 (63%) Female: 3 (38%) Age: 53 ± 13	NA	All patients had chronic massive posterosuperior rotator cuff tear with Goutallier grade 2, 3, or 4 and Hamada grade of 0, 1, or 2 with inability to reduce the rotator cuff to the rotator footprint despite adequate mobilization. No patients had arthritis, and the subscapularis was intact or fully repairable in all cases.	N = 4 (50%)	Four patients had prior rotator cuff repair.
Stone et al, 2021^21^	N = 15 Male: 14 (93%) Female: 1 (7%) Age: 52 ± 7.3 (31-62)	Thirteen patients had occupations classified as labor-intensive.	All patients had irreparable rotator cuff tears determined by preoperative MRI and confirmed intraoperatively. No patients had severe glenohumeral arthritis or Hamada grades 4 and 5 cuff tear arthropathy.	N = 14 (93%)	Fourteen patients had previous rotator cuff repair with an average of 1.8 ± 1.3 (range: 0-5) previous surgeries.
Total	N = 111 Male N = 84 (75.7%) Female N = 27 (24.3%) Age: 55.04 (31-71)	NA	NA	N = 82 (73.9%)	NA

*NA*, not available; *MRI*, magnetic resonance imaging; *SD*, standard deviation.

Data provided as mean ± SD (range).

**Table III:** Functional outcomes across studies.

Study	DASH		ASES		VAS		SANE		SSV (%)	
	Preoperative	Postoperative	Preoperative	Postoperative	Preoperative	Postoperative	Preoperative	Postoperative	Preoperative	Postoperative
Elhassan et al, 2016^8^ (N = 33)	Mean: 52 ± 19 Range: NA	Mean: 18 ± 10 Range: NA	Mean: NA Range: NA	Mean: NA Range: NA	Mean: NA Range: NA	Mean: NA Range: NA	Mean: NA Range: NA	Mean: NA Range: NA	Mean: 54 Range: NA	Mean: 78 Range: NA
Valenti and Werthel, 2018^22^ (N = 14)	Mean: NA Range: NA	Mean: NA Range: NA	Mean: NA Range: NA	Mean: NA Range: NA	Mean: 7 Range: 2-10	Mean: 2 Range: NA	Mean: NA Range: NA	Mean: NA Range: NA	Mean: 40 Range: 10-60	Mean: 70 Range: NA
Elhassan et al, 2020^7^ (N = 41)	Mean: 49 Range: 27-71	Mean: 18 Range: 5-26	Mean: NA Range: NA	Mean: NA Range: NA	Mean: 6 Range: 4-10	Mean: 2 Range: 1-5	Mean: NA Range: NA	Mean: NA Range: NA	Mean: 55 Range: 25-70	Mean: 80 Range: 55-100
Woodmass et al, 2020^24^ (N = 8)	Mean: NA Range: NA	Mean: NA Range: NA	Mean: 56.6 Range: NA	Mean: 84.8 Range: NA	Mean: 2.85 Range: NA	Mean: 1.17 Range: NA	Mean: 34.4 Range: NA	Mean: 68 Range: NA	Mean: NA Range: NA	Mean: NA Range: NA
∗Stone et al, 2021^21^ (N = 12)	Mean: NA Range: NA	Mean: NA Range: NA	Mean: 43.2 Range: NA	Mean: 77.2 Range: NA	Mean: NA Range: NA	Mean: NA Range: NA	Mean: 34.1 Range: NA	Mean: 77.1 Range: NA	Mean: NA Range: NA	Mean: NA Range: NA
Total (N = 108)	N = 74 Mean: 50.34	N = 74 Mean: 18	N = 20 Mean: 48.56	N = 20 Mean: 80.24	N = 63 Mean: 5.8	N = 63 Mean: 1.89	N = 20 Mean: 34.22	N = 20 Mean: 69.86	N = 88 Mean: 52.24	N = 88 Mean: 77.66

*DASH*, Disabilities of the Arm, Shoulder, and Hand; *ASES*, American Shoulder and Elbow Surgeons Score; *VAS*, Visual Analog Scale; *SANE*, Single Assessment Numeric Evaluation; *SSV*, Subjective Shoulder Value; *NA*, not available; *RSA*, reverse total shoulder arthroplasty.

∗ Three of the 15 study participants were excluded from the analysis after undergoing RSA.

**Table IV:** Preoperative and postoperative ROM.

Study	Flexion		External rotation		Abduction	
	Preoperative	Postoperative	Preoperative	Postoperative	Preoperative	Postoperative
Elhassan et al, 2016^8^ (N = 33)	Mean: 70 Range: 20-120	Mean: 120 Range: 80-150	Mean: 20 Range: -50-40	Mean: 47 Range: 10-70	Mean: 40 Range: 20-70	Mean: 90 Range: 60-140
Valenti and Werthel, 2018^22^ (N = 14)	Mean: 150 Range: 100-180	Mean: 160 Range: NA	Mean: -20 Range: -50-0	Mean: 24 Range: NA	Mean: NA Range: NA	Mean: NA Range: NA
Elhassan et al, 2020^7^ (N = 41)	Mean: 67 Range: 30-120	Mean: 133 Range: 90-150	Mean: 25 Range: -50-45	Mean: 70 Range: 20-120	Mean: 50 Range: 20-80	Mean: 95 Range: 65-140
Woodmass et al, 2020^24^ (N=8)	Mean: 101 Range: NA	Mean: 146 Range: NA	Mean: 33 Range: NA	Mean: 44 Range: NA	Mean: NA Range: NA	Mean: NA Range: NA
∗Stone et al, 2021^21^ (N = 12)	Mean: 98 Range: NA	Mean: 144 Range: NA	Mean: 23 Range: NA	Mean: 43 Range: NA	Mean: 74 Range: NA	Mean: 127 Range: 20-120
Total (N = 108)	N = 108 Mean: 85	N = 108 Mean: 135	N = 108 Mean: 18	N = 108 Mean: 52	N = 86 Mean: 50	N = 86 Mean: 98

*ROM*, range of motion; *NA*, not available; *RSA*, reverse total shoulder arthroplasty.

∗ Three of the 15 study participants were excluded from the analysis after undergoing RSA.

**Table V:** Complication rates across included studies.

Study	Total complications	Seroma formation	Hematoma formation	Superficial infection	Deep infection	Tendon rupture	Nerve disruption
Elhassan et al, 2016^8^ (N = 33)	N = 5 (15%)	N = 4 (12%) All resolved spontaneously	N = 0 (0%)	N = 0 (0%)	N = 1 (3%) Infection requiring debridement and later underwent shoulder fusion	N = 0 (0%)	N = 0 (0%)
Valenti and Werthel, 2018^22^ (N = 14)	N = 2 (15%)	N = 0 (0%)	N = 2 (14%) Both had revision surgery for hematoma at harvest site	N = 0 (0%)	N = 1 Patient had *Cutibacterium acnes* infection treated with open debridement and oral antibiotics	N = 0 (0%)	N = 0 (0%)
Elhassan et al, 2020^7^ (N = 41)	N = 10 (23%)	N = 3 (7%) All resolved spontaneously over 2-3 weeks	N = 0 (0%)	N = 1 (2%) Resolved with oral antibiotic treatment	N = 0 (0%)	N = 2 (5%) 2 Shoulders had a traumatic rupture of the transferred tendon with 1 undergoing successful revision aaLTT	N = 4 (9%) All involved hand numbness along the thumb or ulnar nerve distribution. Attributed to postoperative brace with spontaneous resolution 1-3 mo after removing brace.
Woodmass et al, 2020^24^ (N = 8)	N = 0 (0%)	N = 0 (0%)	N = 0 (0%)	N = 0 (0%)	N = 0 (0%)	N = 0 (0%)	N = 0 (0%)
Stone et al, 2021^21^ (N = 15)	N = 3 (20%)	N = 2 (13%) Both underwent superficial wound debridement for seroma and posterior wound dehiscence	N = 0 (0%)	N = 0 (0%)	N = 1 (7%) Patient had cultures positive for *Cutibacterium acnes* at the time of RSA revision surgery	N = 0 (0%)	N = 0 (0%)
Total (N = 111)	N = 20 (18%)	N = 9 (8%)	N = 2 (2%)	N = 1 (1%)	N = 3 (3%)	N = 2 (2%)	N = 4 (4%)

*ROM*, range of motion; *NA*, not available; *aaLTT*, arthroscopy-assisted lower trapezius transfer; *RSA*, reverse total shoulder arthroplasty.

## Results

### Article selection

Using the search strategy outlined above, 362 studies were included for initial screening. After screening through titles and abstracts, 37 full-text citations were reviewed according to the predefined inclusion/exclusion criteria. Five studies encompassing 111 patients met all inclusion criteria and were ultimately included in the scoping review. The study design and surgical technique for each study are outlined in Table I. Patient demographics and rotator cuff injury details from all 5 studies are described in Table II.

### Surgical indications

All 5 studies exclusively included patients with massive irreparable rotator cuff tears as the indication for the LTT transfer surgery (Table II). Four of the 5 studies specifically located massive irreparable rotator tears to the posterosuperior cuff involving the supraspinatus and infraspinatus tendons. Multiple study participants also had concomitant subscapularis and teres minor tears or fatty infiltration. No patients had arthritic changes preoperatively.

### Functional outcomes

Functional outcomes were assessed using The Disabilities of the Arm, Shoulder, and Hand (DASH), Visual Analog Scale (VAS), The American Shoulder and Elbow Surgeons Score (ASES), Single Assessment Numeric Evaluation (SANE), and Subjective Shoulder Value (SSV). The DASH (N = 74), ASES (N = 20), and SANE (N = 20) assessments were reported in 2 studies while the VAS (N = 63) and SSV (N = 88) assessments were reported in 3 studies. The mean change from preoperative to postoperative scores for the DASH (50.34 to 18), ASES (48.56 to 80.24), VAS (5.8 to 1.89), SANE (34.22 to 69.86), and SSV (52.24 to 77.66) were noted. Individual and summative results of functional outcomes across all studies are included in Table III.

### ROM scores

All studies included preoperative and postoperative measures of ROM. Five studies (N = 108) included preoperative and postoperative measures of shoulder flexion and external rotation. Three studies (N = 86) included preoperative and postoperative measures of shoulder abduction. The mean changes from preoperative to postoperative flexion, external rotation, and abduction were 85 to 135, 18 to 52, and 50 to 98, respectively, (Table IV).

### Complications

All studies reported on postoperative complications, with only 1 study reporting the absence of any complication.^24^ Overall, there were a total of 20 complications from a total 111 patients resulting in a general complication rate of 18%. Individual complications rates are provided in Table V, with seroma formation as the most common complication (8%) across all studies.

## Discussion

Rotator cuff surgery is a rapidly evolving branch in orthopedics. While arthroscopic repair represents the most widely used approach to treat rotator cuff lesions, other strategies have been described.^19^ Musculotendinous transfer, initially presented as an experimental technique in 1982 by Robert Cofield (subscapularis transfer)^3^ and in 1988 by Christian Gerber et al (latissimus dorsi transfer),^11^ has since shown increasing promise in select subgroups. These massive irreparable rotator cuff tears may lead to significant patient discomfort and decreased function and ROM of the affected shoulder joint. The LTT transfer technique was initially used in 2014 for patients with paralytic shoulders and loss of external rotation.^6^ It has since been applied in the setting of massive irreparable rotator cuff tears, starting in 2016.^8^ Our study showed that the LTT technique improved patients’ shoulder pain and postoperative function and ROM for irreparable rotator cuff tears with an overall complication rate of 18%. Compared to other surgical options, the LTT may be best suited in young active patients lacking glenohumeral arthritic changes since tendon transfers do not address arthritic changes in the glenohumeral joint.^10^ Currently, the decision to perform either the latissimus dorsi tendon (LDT) or the LTT transfer is based on surgeon preference given the limited data showing superiority of either procedure.^10^ One biomechanical study showed that patients with external rotation as the main functional deficit may benefit more from an LTT transfer than from an LDT transfer; conversely, a patient whose main deficit is external rotation with the arm at 90° of abduction would benefit more from an LDT transfer.^13^ Another biomechanical study showed LTT transfer to be superior to LDT transfer at restoring native glenohumeral kinematics and joint reaction forces.^16^

The surgical anatomy of LTT transfers has been determined to offer safe and reliable anatomic relationships for transfer with no direct neurovascular injury according to a study on 10 cadaveric specimens.^15^ Omid et al suggest that dissection medial to the tip of the tendinous portion of the lower trapezius can be performed to a minimum of approximately 23 mm and on average 58 mm without encountering the spinal accessory nerve.^15^ Additionally, Ghoraishian et al have suggested that improved techniques such as mini-open and arthroscopic assisted approaches using a horizontal incision for tendon harvest can avoid damage to the accessory nerve and problems such as acromial nonunion, thereby limiting complications.^12^

In all studies we reviewed, participants lacked preoperative severe glenohumeral arthritic changes as the criterion for LTT transfer consideration. Additionally, concomitant subscapularis tears are not contraindications for LTT transfers because the trapezius contracts during external rotation.^8^ This is not reproduced with the LDT transfer (LDT-T) technique, and concomitant subscapularis tears had been shown to have poorer functional outcomes with the LDT-T procedure.^10^ Elhassan et al included 20 patients with partial subscapularis tears and found no difference in postoperative outcomes between patients with and those without preoperative subscapularis tears undergoing an LTT transfer.^7^ However, they identified negative clinical outcomes in patients having advanced rotator cuff arthropathy changes and shoulder pseudoparalysis.

There were significant differences in the surgical technique between studies included in the review. Four of the 5 studies^7^^,^^8^^,^^21^^,^^24^ used an Achilles tendon allograft while 1 study^22^ used a semitendinosus autograft for graft augmentation. Due to limited data, no analyses between graft types could be completed. Most studies performed the LTT transfer using an arthroscopic approach^7^^,^^21^^,^^22^^,^^24^ while 1 study^8^ used solely an open approach. The suggested benefits of using an arthroscopically assisted approach include the minimally invasive nature of the procedure. Irrespective of the surgical approach, all studies demonstrated increased functional outcomes and ROM postoperatively with the LTT procedure. We identified the following preoperative to postoperative improvements in mean ROM with LTT transfer for flexion (85 to 135), external rotation (18 to 52), and abduction (50 to 98). Improvements in functional outcomes were evident across the DASH, ASES, VAS, SANE, and SSV assessments. Only 1 study directly compared arthroscopic LTT transfer to the LDT-T and found the LTT transfer provided significantly improved functional outcomes at 2 years postoperatively.^24^

Across all studies included in this review, no complications were identified as being related to damage to the spinal accessory nerve. The overall complication rate was 18% across all studies, with seroma formation being the most common postoperative complication. Of note, 2 patients (2%) had tendon rupture postoperatively with 3 patients (3%) developing deep infections. Although no direct comparison for complications was made between LTT transfer and other surgical techniques, the complication rates outlined above are consistent with those in a systematic review for reverse shoulder arthroplasty in treating massive irreparable rotator cuff injuries.^17^ Specifically, 4 patients receiving LTT did not show any clinical improvement in the study by Elhassan et al.^7^ Three of these patients had arthritic changes preoperatively, and 1 had significant pseudoparalysis preoperatively. Of the 4 patients with Hamada grade 2 or greater arthritic changes, only 2 had functional improvements, with the other 2 patients requiring a subsequent reverse shoulder arthroplasty for symptom relief. These findings are consistent with the general knowledge of using the LTT in young patients without glenohumeral arthritis since the LTT technique provides no benefit for treating glenohumeral arthritis.

### Limitations

There are several limitations with our review. Due to the novelty of the LTT transfer in treating massive irreparable rotator cuff tears, we only identified 5 studies encompassing 111 patients to assess postoperative outcomes. Studies differed in what scores (DASH, ASES, VAS, SANE, SSV) were used to assess postoperative function, limiting the sample size for each individual metric in our review. Four studies in our review were retrospective case studies, with 1 prospective case series that may have introduced inherent bias into our results. The lack of randomized controlled trials limited our ability to compare the LTT transfer to other surgical techniques for the treatment of massive irreparable rotator cuff tears. Another limitation of this study is the heterogeneity of the surgical techniques across each of the 5 included studies. There were differences in approach (open LTT vs. arthroscopically assisted LTT), graft type (Achilles vs. semitendinosus), and materials (suture anchors vs. buttons). These differences may have contributed to the heterogeneity in the mean total values reported across all studies and limited the external validity of this study. Furthermore, conclusions about outcomes and complications from LTT may vary depending on specific surgical techniques. We were not able to analyze differences between surgical techniques with this review. Due to the limited number of studies and small sample size, we were unable to analyze how differences in surgical technique and preoperative patient functional status impacted postoperative outcomes. Additional sources of heterogeneity which may have affected outcomes include surgeon experience, differences in the postoperative rehabilitation therapy protocol, patients' baseline demographics, and follow-up duration among others. Due to the limited number of studies and high variation between studies, a meta-analysis was not possible. More research, preferably in the form of randomized controlled trials, is necessary to perform a meta-analysis and have greater confidence in the postoperative functional outcomes and complications than the preliminary data presented here. Additionally, comparing different surgical techniques (arthroscopic vs. open), preoperative patient functional status, and allograft types in future studies may further elucidate the optimal practices for using the LTT technique in the setting of irreparable rotator cuff injuries.

## Conclusion

Our analysis showed that LTT transfer improved postoperative function, ROM, and pain for patients with irreparable rotator cuff tears with an overall complication rate of 18%. Future controlled studies are required to directly compare LTT transfer to other tendon transfers and other surgical techniques for irreparable rotator cuff tears.

## Disclaimers:

Funding: No funding was disclosed by the authors.Conflicts of interest: The authors, their immediate families, and any research foundation with which they are affiliated have not received any financial payments or other benefits from any commercial entity related to the subject of this article.

## References

[1] Aibinder W.R., Elhassan B.T. (2018). Lower trapezius transfer with Achilles tendon augmentation: indication and clinical results. Obere Extremität.

[2] Clouette J., Leroux T., Shanmugaraj A., Khan M., Gohal C., Veillette C. (2020). The lower trapezius transfer: A systematic review of biomechanical data, techniques, and clinical outcomes. J Shoulder Elbow Surg.

[3] Cofield R.H. (1982). Subscapular muscle transposition for repair of chronic rotator cuff tears. Surg Gynecol Obstet.

[4] Cvetanovich G.L., Waterman B.R., Verma N.N., Romeo A.A. (2019). Management of the irreparable rotator cuff tear. J Am Acad Orthop Surg.

[5] Dwyer T., Razmjou H., Henry P., Gosselin-Fournier S., Holtby R. (2015). Association between pre-operative magnetic resonance imaging and reparability of large and massive rotator cuff tears. Knee Surg Sports Traumatol Arthrosc.

[6] Elhassan B. (2014). Lower trapezius transfer for shoulder external rotation in patients with paralytic shoulder. J Hand Surg.

[7] Elhassan B.T., Sanchez-Sotelo J., Wagner E.R. (2020). Outcome of arthroscopically assisted lower trapezius transfer to reconstruct massive irreparable posterior-superior rotator cuff tears. J Shoulder Elbow Surg.

[8] Elhassan B.T., Wagner E.R., Werthel J.D. (2016). Outcome of lower trapezius transfer to reconstruct massive irreparable posterior-superior rotator cuff tear. J Shoulder Elbow Surg.

[9] Gerber C., Fuchs B., Hodler J. (2000). The results of repair of massive tears of the rotator cuff. J Bone Joint Surg Am.

[10] Gerber C., Rahm S.A., Catanzaro S., Farshad M., Moor B.K. (2013). Latissimus dorsi tendon transfer for treatment of irreparable posterosuperior rotator cuff tears: long-term results at a minimum follow-up of ten years. J Bone Joint Surg Am.

[11] Gerber C., Vinh T.S., Hertel R., Hess C.W. (1988). Latissimus dorsi transfer for the treatment of massive tears of the rotator cuff. A preliminary report. Clin Orthop Relat Res.

[12] Ghoraishian M., Stone M.A., Elhassan B., Abboud J., Namdari S. (2020). Techniques for lower trapezius tendon transfer for the management of irreparable posterosuperior rotator cuff tears. J Orthop.

[13] Hartzler R.U., Barlow J.D., An K.N., Elhassan B.T. (2012). Biomechanical effectiveness of different types of tendon transfers to the shoulder for external rotation. J Shoulder Elbow Surg.

[14] Lädermann A., Denard P.J., Collin P. (2015). Massive rotator cuff tears: definition and treatment. Int Orthop.

[15] Omid R., Cavallero M.J., Granholm D., Villacis D.C., Anthony M.Y. (2015). Surgical anatomy of the lower trapezius tendon transfer. J Shoulder Elbow Surg.

[16] Omid R., Heckmann N., Wang L., McGarry M.H., Vangsness C.T., Lee T.Q. (2015). Biomechanical comparison between the trapezius transfer and latissimus transfer for irreparable posterosuperior rotator cuff tears. J Shoulder Elbow Surg.

[17] Petrillo S., Longo U.G., Papalia R., Denaro V. (2017). Reverse shoulder arthroplasty for massive irreparable rotator cuff tears and cuff tear arthropathy: a systematic review. Musculoskelet Surg.

[18] Ramme A.J., Robbins C.B., Patel K.A., Carpenter J.E., Bedi A., Gagnier J.J. (2019). Surgical versus nonsurgical management of rotator cuff tears: a matched-pair analysis. J Bone Joint Surg Am.

[19] Randelli P., Cucchi D., Ragone V., de Girolamo L., Cabitza P., Randelli M. (2015). History of rotator cuff surgery. Knee Surg Sports Traumatol Arthrosc.

[20] Ryösä A., Laimi K., Äärimaa V., Lehtimäki K., Kukkonen J., Saltychev M. (2017). Surgery or conservative treatment for rotator cuff tear: a meta-analysis. Disabil Rehabilx.

[21] Stone M.A., Kane L.T., Ho J.C., Namdari S. (2021). Short-term outcomes of lower trapezius tendon transfer with Achilles allograft for irreparable posterosuperior rotator cuff tears. Arthrosc Sports Med Rehabil.

[22] Valenti P., Werthel J.D. (2018). Lower trapezius transfer with semitendinosus tendon augmentation. Obere Extremität.

[23] Wagner E.R., Woodmass J.M., Welp K.M., Chang M.J., Elhassan B.T., Higgins L.D. (2018). Novel arthroscopic tendon transfers for posterosuperior rotator cuff tears: latissimus dorsi and lower trapezius transfers. JBJS Essent Surg Tech.

[24] Woodmass J.M., Wagner E.R., Chang M.J., Welp K.M., Grubhofer F., Higgins L.D. (2020). Arthroscopic lower trapezius tendon transfer provides equivalent outcomes to latissimus dorsi transfer in the treatment of massive posterosuperior rotator cuff tears. J ISAKOS.

[25] Yamamoto A., Takagishi K., Osawa T., Yanagawa T., Nakajima D., Shitara H. (2010). Prevalence and risk factors of a rotator cuff tear in the general population. J Shoulder Elbow Surg.

[26] Yoo J.C., Ahn J.H., Yang J.H., Koh K.H., Choi S.H., Yoon Y.C. (2009). Correlation of arthroscopic repairability of large to massive rotator cuff tears with preoperative magnetic resonance imaging scans. Arthroscopy.

