# Supplementary material for: Lower trapezius tendon transfer for irreparable rotator cuff injuries: a scoping review
Source: JSES Rev Rep Tech. 2022 Sep 30;3(1):1–9. doi: 10.1016/j.xrrt.2022.08.006 (PMC10426520; doi:10.1016/j.xrrt.2022.08.006)
Supplement: Supplementary Appendix S1 [file mmc1.docx]

**Appendix 1.** Search Algorithm

Search completed on January 21st, 2022

Ovid Medline(R) 1946 to present: Results

1. trapezius.mp 4644
2. transfer.mp 561739
3. 1 and 2 244

Search completed on January 21st, 2022

Embase Classic + Embase 1947 to present: Results

1. trapezius.mp 7496
2. transfer.mp 705416
3. 1 and 2 307
